# Exploring the intersection of racism, antimicrobial resistance, and vaccine equity

**DOI:** 10.1017/ash.2022.283

**Published:** 2022-08-05

**Authors:** Jacinda C. Abdul-Mutakabbir, Brenda Simiyu

**Affiliations:** 1 Department of Pharmacy Practice, Loma Linda University School of Pharmacy, Loma Linda California; 2 Department of Basic Sciences, Loma Linda University School of Medicine, Loma Linda California; 3 Department of Pharmacy Services, University Medical Center, New Orleans, Louisiana

## Abstract

Structural racism and systemic health inequities have an overwhelming and deadly impact on racially and ethnically minoritized groups. Antimicrobial resistance (AMR) is widely considered a global public health threat, and concerns that minoritized groups are disproportionately affected are increasing. With the emergence and spread of AMR, novel therapies and prevention strategies are imperative. Coronavirus disease-19 (COVID-19) has highlighted stark imbalances in the hospitalization and death rates of minoritized individuals compared to their White counterparts, irrespective of the availability of targeted preventive therapies (ie, vaccinations). Thus, dialogue regarding the utility of vaccines used prophylactically to decrease the number of infectious diseases cases and the historical lack of vaccine equity and uptake across minoritized groups is needed. All of these factors work in concert to increase the burden of AMR and ultimately health disparities within minoritized communities. Herein, we provide historical context pertaining to the impact of structural racism on healthcare inequities in the United States, we explore racial and ethnic disparities in AMR, and we discuss the intersection of racism, AMR, and vaccine equity. Lastly, we offer recommendations to mitigate the described inequities.

Profound racial and ethnic disparities are deeply woven within the constructs of the US healthcare system.^1,2^ These inequities are largely explained by racism and its effects on the lives of racially and ethnically minoritized individuals.^1,2^ More importantly, racism is independently related to all structural and social determinants of health.^3^ Structural factors, including the social separation of racial groups, through segregating policies, introduce environmental pollutants and infectious agents into minoritized communities.^1–3^ Additionally, racism within systems and policies, has resulted in lower rates of racial and ethnic minoritized groups that enroll or persist in higher education, limiting their employment opportunities and often translating to their lower socioeconomic status (SES).^1–3^ Inevitably, lower SES results in the reduced rates of health insurance and access to healthcare presently recognized across minoritized groups.^1–3^ Consequently, these individuals remain at increased risk for ill or unmanaged, acute, and chronic comorbidities.^4,5^ Infectious diseases are the second leading contributor to healthcare racial disparities, and the management of these morbidities often requires the use of antimicrobial therapy.^6,7^

As infectious diseases have evolved and antimicrobial utilization has increased, the rapid propagation and development of advanced mechanisms of resistance have become a substantial clinical problem.^8^ Antimicrobial resistance (AMR) is a public health threat and is widely considered a global pandemic.^9,10^ Furthermore, >100,000 people die from multidrug-resistant infections each year, positioning AMR as a leading cause of death.^9,10^ Notably, efforts to reduce AMR have included the development of national resistance surveillance databases and novel antimicrobial agents, the use of rapid diagnostics tools to quickly identify resistant organisms, and preventive therapeutics (vaccinations) to decrease disease burden. However, research that explores the racial disparities in antimicrobial usage and AMR across racial and ethnic groups is lacking.^11,12^

## Racial disparities in antimicrobial use and resistance in healthcare settings

Currently there are conflicting reports on whether racial differences and/or disparities exist in antimicrobial usage, prescribing patterns, and resistance trends.^10,13^ Nonetheless, among the few studies that do report racial inequities in antimicrobial usage, those observed in intensive care units (ICU) bring forth several interesting factors.^14^ Notably, ICUs have been the focal points for the emergence and spread of AMR pathogens.^14,15^ Minoritized patients, specifically non-Hispanic Black individuals are less likely to receive timely antimicrobials for the treatment of severe morbidities including sepsis and septic shock in the ICU.^16^ Of note, the time to appropriate antimicrobial is independently associated with ICU patient mortality because the causative organisms isolated are often characterized by multidrug resistance.^14,15,17^ Moreover, the delay in appropriate therapy can result in the continued growth, propagation, and widespread dissemination of the resistant isolates.^15^

Interestingly, hospital system and/or setting has been shown to be a factor in the quality of ICU care, and ultimately in the receipt of appropriate and timely antimicrobial therapy.^14,18^ When treated in hospitals with equal access to care, such as Veterans’ Affairs health systems, minoritized and non-Hispanic White patients have similar outcomes and access to essential treatments including antimicrobials, which may explain the lessened racial differences observed in AMR rates in this setting.^18^ However, minoritized patients are more likely to frequent hospitals in areas of lower SES, where funding and treatment resources are often minimal.^14,19,20^

The lack of access to adequate resources and care, due to SES classification, resulting in disparities in AMR infection rates, has been described by See et al.^21^ These researchers reported that neighborhoods characterized by lower SES, and populated by minoritized individuals, had a higher incidence of invasive community-associated MRSA (CA-MRSA) infections.^21^Although antimicrobial regimens for the treatment of CA-MRSA were not described in this study, other investigators have uncovered racial disparities in the management of skin and soft-tissue infections (SSTIs) caused by CA-MRSA.^22^ Non-Hispanic Black inpatients were more like to be prescribed clindamycin and not cefazolin therapy (the first-line option) than non-Hispanic White inpatients.^22^ This situation is troublesome because clindamycin requires frequent dosing and is associated with *Clostridioides difficile* infection (CDI) where racial disparities in incidence rates and clinical management have also been described.^6,23^ Even though data regarding income, SES, and insurance status or type were not collected, they are often factors in decisions regarding treatment selection.^1,3^

Nonetheless, racial disparities in the incidence and prevalence of AMR infections and poor prescribing patterns for the prophylaxis and/or treatment of infectious morbidities is not specific to gram-positive organisms, adult patients, or inpatient treatment settings. In a retrospective study conducted at the Detroit Medical Center (DMC), investigators reported a greater number of carbapenem-resistant Enterobacteriaceae bloodstream infections in non-Hispanic Black patients than in non-Hispanic White patients.^24^ Although SES and other sociodemographic factors were uncollected, the DMC is located in Detroit, Michigan, where Black individuals comprise >70% of the population and >30% of the city’s population is impoverished.^25^

Of interest, Goyal et al^26^ reported that non-Hispanic Black and Hispanic or Latino children were less likely to receive antimicrobials than their non-Hispanic White counterparts in the treatment of viral acute respiratory illnesses. These researchers linked this racial difference to potential provider implicit biases and to the historical overtreatment of non-Hispanic White patients due to their perceived increase in disease severity compared to minoritized patients.^26^ Nonetheless, antimicrobial prescribing practices for dentists in the United States are opposite those reported by Goyal et al.^27^ A retrospective study revealed that non-Hispanic Black and Hispanic or Latino patients respectively had 21% and 6% higher likelihoods of receiving antimicrobials following a dental visit compared to their non-Hispanic White counterparts.^27^ These researchers hypothesized that this was because the minoritized patients often required advanced procedures due to lack of regular access to dental care.^27^ Notably, several socioeconomic factors, including insurance status and SES, were identified as predictive markers of antimicrobial receipt.^27^ In sum, these studies show the impact that structural racism resulting in low SES, as well as provider biases, can have on AMR prescribing patterns and the potential for deleterious outcomes within minoritized communities.

## Minimal resources to combat antimicrobial resistance in minoritized communities

To mitigate the rising rates of AMR potentiated by the misuse of the antimicrobials, the federal government created a National Task Force on Combatting Antibiotic-Resistant Bacteria (CARB).^12^ This task force released a plan to improve antimicrobial use that included accelerating research on novel therapeutics and optimizing antimicrobial stewardship programs and requirements.^12^ Despite the plan’s focus on collecting and utilizing data to better understand resistance patterns, racial and ethnic disparities and associated outcomes are not recognized priorities.^12^

Formidable challenges remain in reaching the outlined goals and objectives of the plan within minoritized communities. Irrespective of the recognized need for novel therapeutics, new, innovative antimicrobials in the clinical pipeline are scarce, specifically those targeting extensively or multidrug-resistant organisms.^28^ Due to the limited supply, the allocation of the available novel agents is often limited to hospitals and institutions that can accommodate the costs.^29^ Consequently, the hospitals located in areas of lower SES, which minoritized patients are more likely to frequent, are less likely to be able to afford the heavily sought after but limited therapeutics.^30^

Additionally, poorly funded hospitals are also less likely to have the resources to employ the key stakeholders necessary for successful antimicrobial stewardship programs.^31,32^ This personnel includes trained infectious diseases physicians and pharmacists who can educate general providers and reduce the selection for AMR. These hospitals may also be less likely to have the ability to quickly identify prominent drug-resistant gene markers to optimize antimicrobial selections.^33^ Rapid diagnostic tools that are designed to quickly identify select and predetermined resistance genes have been shown to significantly enhance antimicrobial stewardship programs and improve AMR rates.^34^ Nevertheless, such technologies are typically associated with high costs; thus, the likelihood of their placement in hospitals largely populated by minoritized individuals is low.^32,33^

## The impact of vaccination inequities on antimicrobial resistance

In addition to novel therapies and antimicrobial stewardship programs, CARB has also highlighted the important role that vaccinations have in decreasing AMR.^12^ Because they are used to prevent infectious disease, they are also vital to the prevention of subsequent secondary bacterial infections.^11^ Irrespective of the known role of vaccines in the prevention of respiratory infectious diseases and AMR, non-Hispanic Black, Hispanic/Latino, and Native American individuals are less likely to be immunized than White persons.^35,36^ As a result of this lack of uptake, the Centers for Disease Control (CDC) reports that these individuals are historically more likely to be hospitalized for extended intervals due to preventable respiratory infectious diseases.^36^ In part, this can be attributed to the systemic barriers that force minoritized individuals into multigeneration and high-density living arrangements, which can increase disease transmission.^1,2^ Also, minoritized individuals are more likely to have comorbidities that further complicate their disease severity and may compromise their humoral immune response functions.^4,5,37^ Furthermore, severe illness and complications, leading to prolonged hospital lengths of stay, have been linked to the development of AMR infections secondary to the primary infectious disease (viral infection).^38^ More recently, hospitalizations due to severe coronavirus disease-19 (COVID-19) have been shown to result in the development of AMR infections.^39^ Despite the lack of data describing and quantifying the impact of secondary infections on patient outcomes, and whether racial differences exist, history attests to the likelihood of significant disparities.

The lack of vaccine uptake across minoritized groups for respiratory infectious diseases, specifically COVID-19, has been linked to several factors including hesitancy due to systemic racism and decades of medical mistreatment by the federal government as well as the vaccine inequities within minoritized communities.^40–42^ Trustworthy messaging from racially concordant providers remains integral to increasing vaccine confidence and subsequently the willingness to be vaccinated.^40,42,43^ Nevertheless, for those individuals who are interested in being immunized, the inequitable distribution of the vaccines and lack of access within minoritized communities in the United States are clear limitations.^43,44^ Minoritized individuals are more likely to live in healthcare-provider and pharmacy deserts with limited locations where they can receive immunizations.^19^

Furthermore, registration processes for the vaccines are largely Web based; however, minoritized communities are less likely than their White counterparts to have Internet access as well as e-mail accounts necessary for registration completion.^45,46^ Language barriers and inaccurate translation on health department registration websites are also limitations to vaccination^47^ that widen the digital divide and its impact on equitable healthcare in the United States.^45^ Furthermore, minoritized individuals often rely on mass-transit modalities, but many of the available vaccination sites are in locations where these transportation mechanisms are not accessible.^45^ Additionally, vaccination schedules often conclude during early evening hours, which creates a barrier for essential working personnel.^47^

Although strategies were rapidly developed at the start of the COVID-19 vaccination roll-out to address these limitations, including the establishment of low-barrier community-based vaccination clinics, many of these efforts have ceased as funding sources have evaporated.^43^ Consequently, >1 year following the availability of the COVID-19 vaccines, >50% of minoritized individuals remain unvaccinated.^48^ Therefore, they are at heightened risk for severe COVID-19 and prolonged hospital visits for adequate treatment.^49^ Thus, the risk of AMR resistance, due to infection by a vaccine-preventable disease, remain ever present for minoritized individuals.

## Recommendations for addressing the intersection of racism, antimicrobial resistance, and vaccine equity

**Fig. 1.**
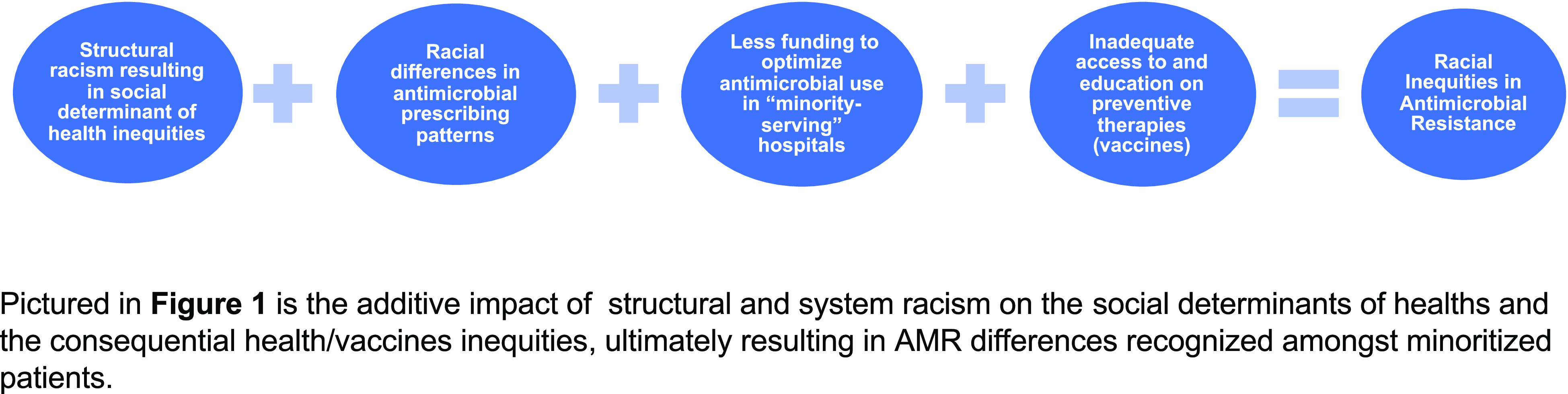
The additive impact of structural racism on antimicrobial resistance and vaccine inequities.

Despite the limited, and in some ways, conflicting data that exist describing racial differences in AMR, it remains clear that socioeconomic and demographic factors, influenced by racism, affect the reported disparities (Fig. 1). Thus, exploring research and clinical applications to fully understand all the factors that contribute to racial or ethnic disparities in AMR is imperative. We recommend several action points to address the known aspects of AMR racial differences and others to rectify the existing knowledge gaps on the subject.

1. We must acknowledge that inequities in social determinants of health, as well as provider implicit and explicit biases, heavily influence antimicrobial prescribing patterns and the propagation of AMR. The lack of access to adequate healthcare is a consistent and significant contributor to the observed racial differences in prescribing patterns, resistance trends, and receipt of preventive therapies. It is imperative that healthcare professionals, identify their role in addressing and mitigating access limitations within minoritized communities. To effectively do this, they must identify, confront, and dismantle their own racial biases because they heavily influence clinical decision making. Healthcare professionals must also hold their colleagues accountable for any biased actions or attitudes that may negatively affect minoritized patients.
2. We must design research that accurately quantifies and characterizes existing racial differences and/or inequities in AMR. Race and ethnicity demographic data are often absent from AMR-related studies, despite recent federal efforts to improve the availability of this information.^12^ The inclusion of this information and an exploration of the nuances in AMR within minoritized groups would be a worthwhile undertaking for researchers. The alteration of each goal and objective of the CARB national action plan to include the collection of specific race and ethnicity data to quantify differences is critical. As of March 2022, the Centers for Disease Control has expressed a commitment to facilitating the regular collection and reporting of health inequities related to key bacterial pathogens as well as other sociodemographic data (eg, SES, etc) that would holistically identify mediating factors in the observed AMR inequities.^50^ Cross sharing of information and collaboration among these groups and the shared prioritization of identifying racial disparities in AMR will increase the likelihood of change.
3. In vitro research that explores racial differences in the genotypes, phenotypes, or host immune factors that may contribute to AMR is needed as we navigate the best methods to equitably improve patient outcomes. Research quantifying the use of rapid diagnostics tools and their placement in hospitals that serve primarily minoritized communities is needed. Nonetheless, to adequately address AMR health equity gaps, it will be essential to provide and allocate federal funding to investigate researching and reporting opportunities.
4. We must advocate for increased funding to advance AMR interventions and strategies within “minority-serving” institutions as well as the equitable pricing of novel technologies and therapeutics. Because minimal financial resources are a limitation to AMR management within minoritized communities, this additional funding would give hospitals in these areas the ability to employ specialized clinicians (physicians, pharmacists, microbiologists) to develop robust antimicrobial stewardship teams that will aid in promoting positive patient outcomes. Equitable pricing of rapid diagnostic tools would allow institutions to further optimize their stewardship programs. Additionally, antimicrobial pricing that unlinks revenues from sales would make novel therapies more accessible for institutions and would contribute to pharmacoequity.
5. We must recognize the role of vaccine equity in decreasing AMR within minoritized communities. Promoting vaccine equity and increased uptake among racially and ethnically minoritized groups is essential. Vaccines must be specifically allocated by federal, state, and local governments to vulnerable minoritized communities. Following allocation, developing innovative strategies to increase vaccine confidence will be important for raising immunization rates. Targeted education efforts in nontraditional community settings that include trusted faith and community leaders can be successful in changing attitudes surrounding vaccines across minoritized groups.^40,43^ The role of immunizations in overcoming AMR should also be included in the education provided to communities to increase health literacy. Finally, interventions that include the placement of low-barrier vaccination clinics, alongside direct allocation and education, should be prioritized because they are substantially effective in increasing vaccination uptake and potentially reducing AMR disparities.^43^

In conclusion, structural racism has a longstanding history in the United States, and it permeates almost every system, including health care. The AMR global pandemic has the potential to severely affect minoritized groups. Therefore, we must be intentional in identifying racial differences in antimicrobial usage, prescribing, and characterized genotypes and phenotypes to effectively create strategies to overcome the inequities. As cornerstones, these strategies should include initiatives to promote vaccine equity within minoritized communities, to advance health literacy on AMR, and to establish easily accessible opportunities for vaccination. Finally, we must advocate for funding to be specifically allocated to institutions that primarily serve minoritized groups so they have access to novel medications and diagnostics tools and can employ the necessary personnel to effectively reduce AMR. Overall, a proactive approach is necessary to address the factors that contribute to AMR racial inequities.
